# Supplementary material for: Transcriptome Profile Analysis of Winter Rapeseed (Brassica napus L.) in Response to Freezing Stress, Reveal Potentially Connected Events to Freezing Stress
Source: Int J Mol Sci. 2019 Jun 5;20(11):2771. doi: 10.3390/ijms20112771 (PMC6600501; doi:10.3390/ijms20112771)
Supplement: Supplementary file 1 [file ijms-20-02771-s001.zip › 5.29 addition file/Figure S11.pdf]

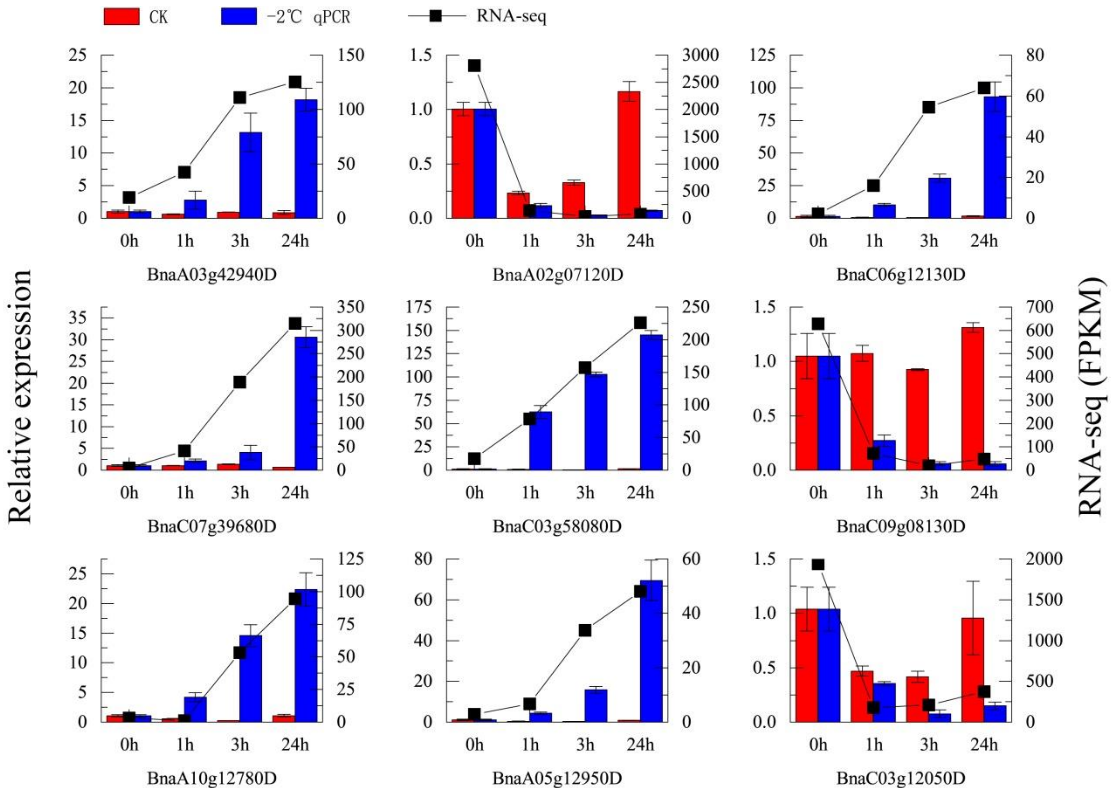

Figure S11 qRT-PCR analysis of selected DEG genes in *Brassica napus* of non-stressed (CK) and freezing-stressed (-2℃). Error bars represent standard errors of the relative expression levels mean values by qRT-PCR (n=4) (left y-axis). Point plot represent transcript levels change (log2 FC) according to the FPKM value of RNA-Seq (right y-axis).
