# Supplementary figures and images for: Transcriptome Profile Analysis of Winter Rapeseed (Brassica napus L.) in Response to Freezing Stress, Reveal Potentially Connected Events to Freezing Stress
Source: Int J Mol Sci. 2019 Jun 5;20(11):2771. doi: 10.3390/ijms20112771 (PMC6600501; doi:10.3390/ijms20112771)

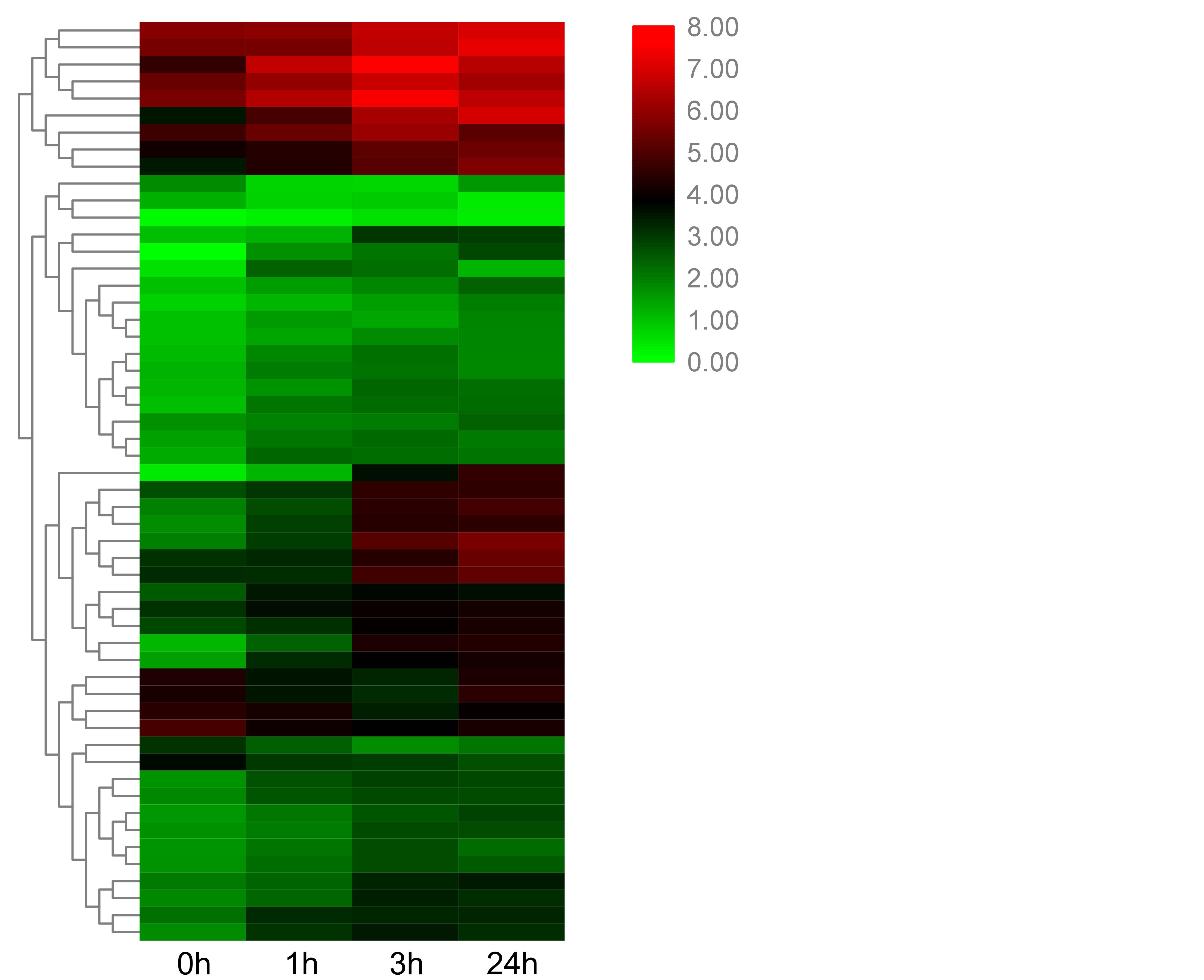

Supplement: Supplementary file 1 [file ijms-20-02771-s001.zip › 5.29 addition file/Figure S10_Heat map of DEGs enriched in Environmental adaptation.xlsx.jpg]

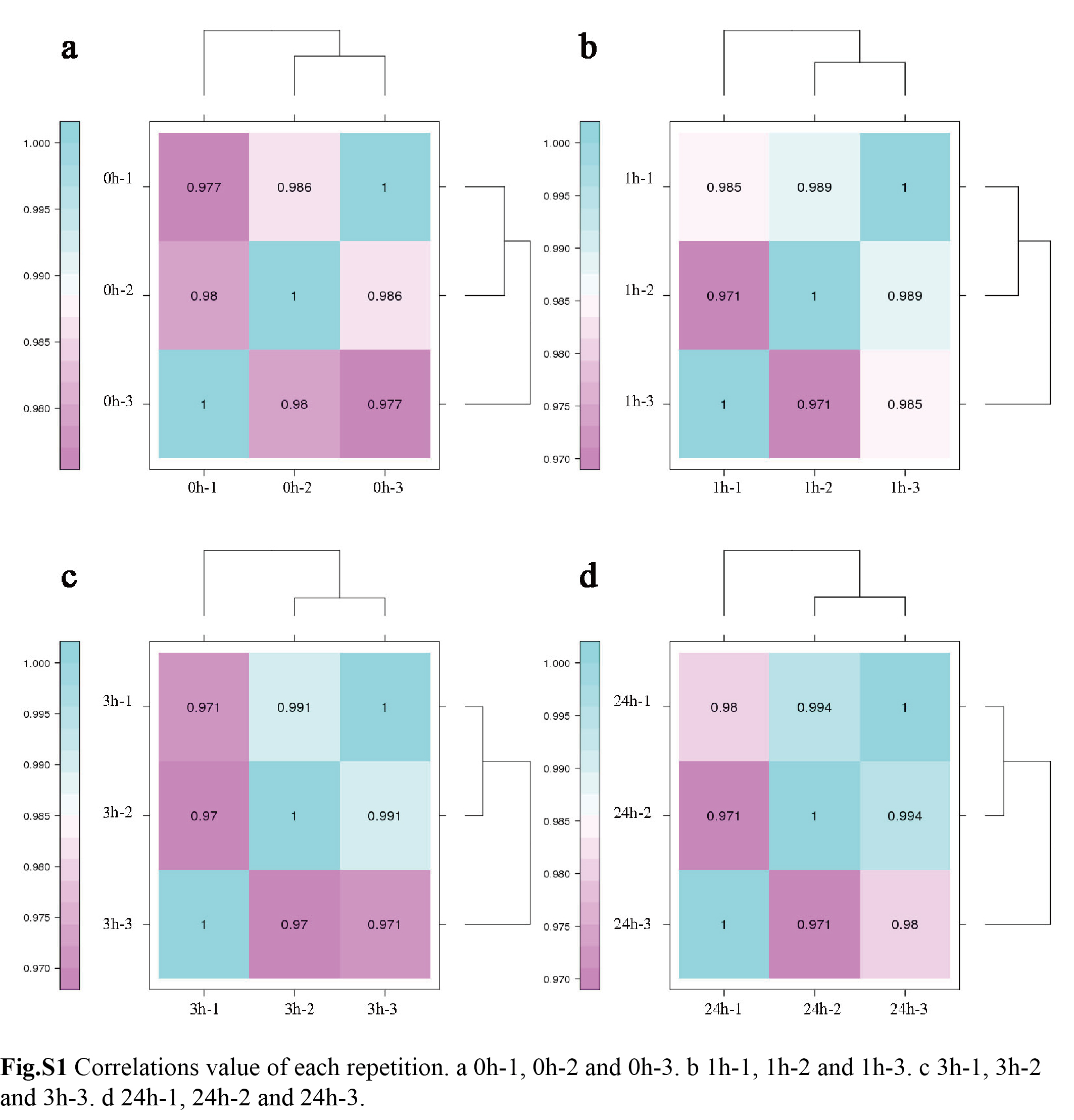

Supplement: Supplementary file 1 [file ijms-20-02771-s001.zip › 5.29 addition file/Figure S1_correlations value of each repetition.jpg]

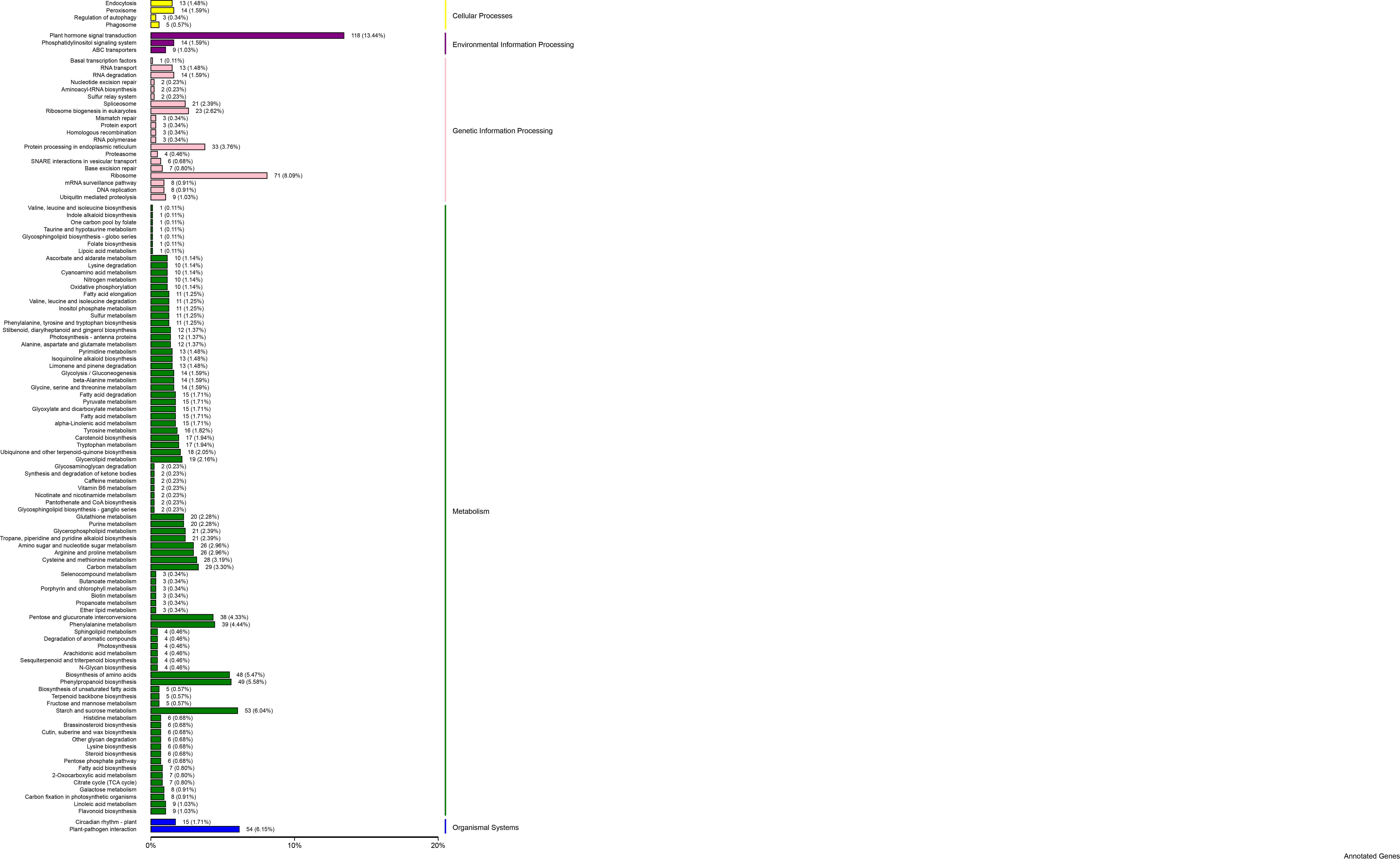

Supplement: Supplementary file 1 [file ijms-20-02771-s001.zip › 5.29 addition file/Figure S2_KEGG classification of all DEGs..jpg]

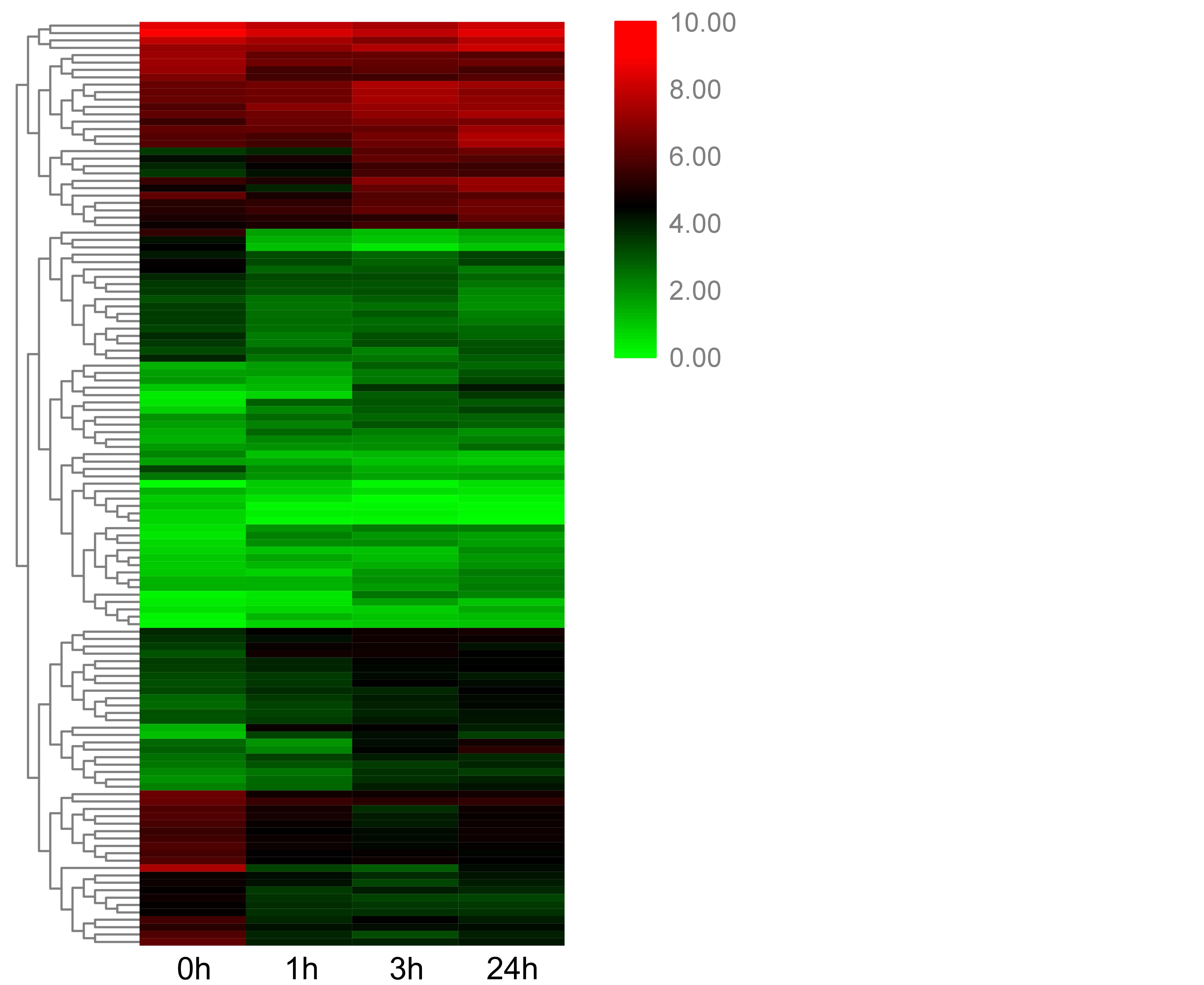

Supplement: Supplementary file 1 [file ijms-20-02771-s001.zip › 5.29 addition file/Figure S3_Heat map of DEGs enriched in Amino acid metabolism pathway .xlsx.jpg]

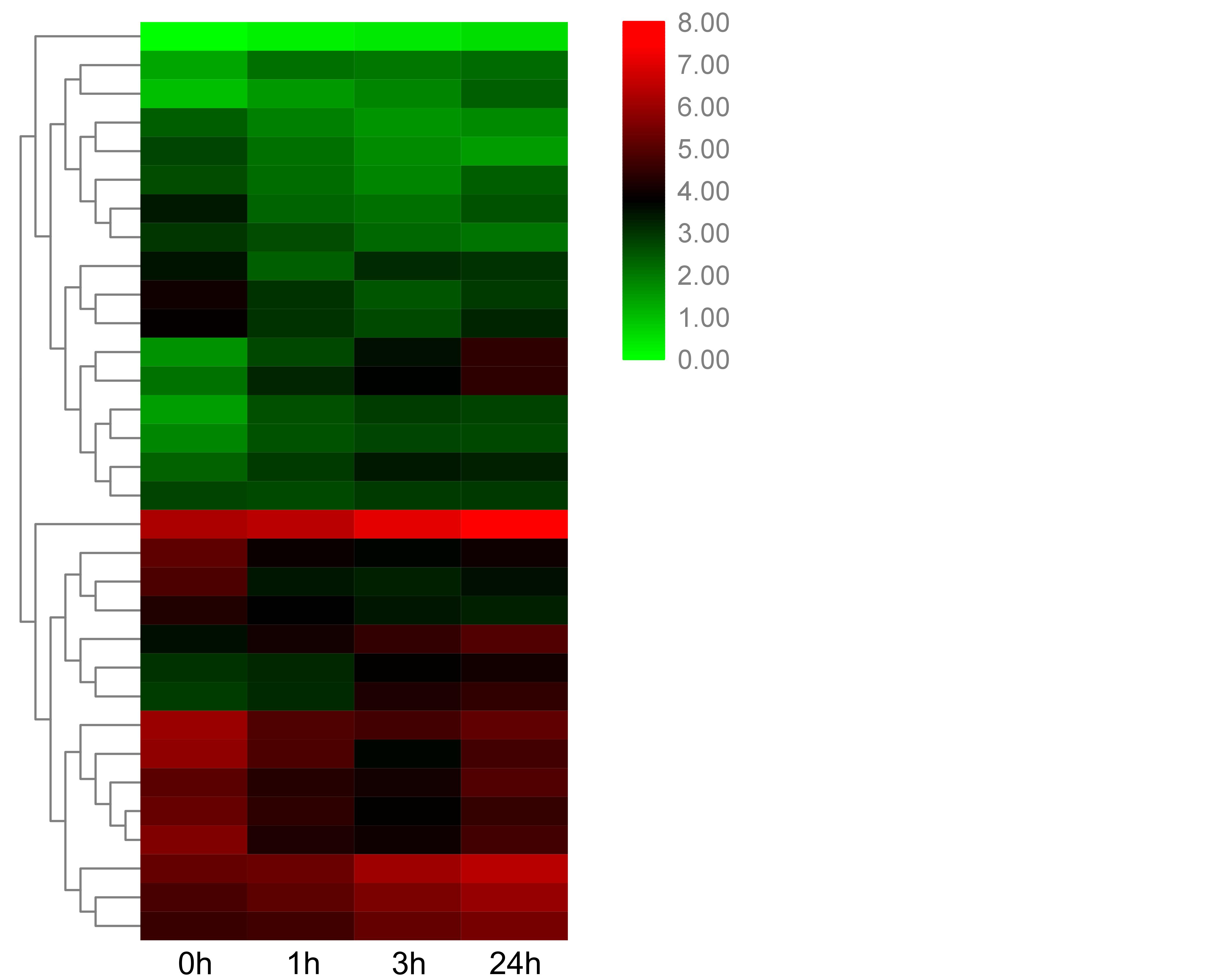

Supplement: Supplementary file 1 [file ijms-20-02771-s001.zip › 5.29 addition file/Figure S4_Heat map of DEGs enriched in Lipid metabolism.xlsx.jpg]

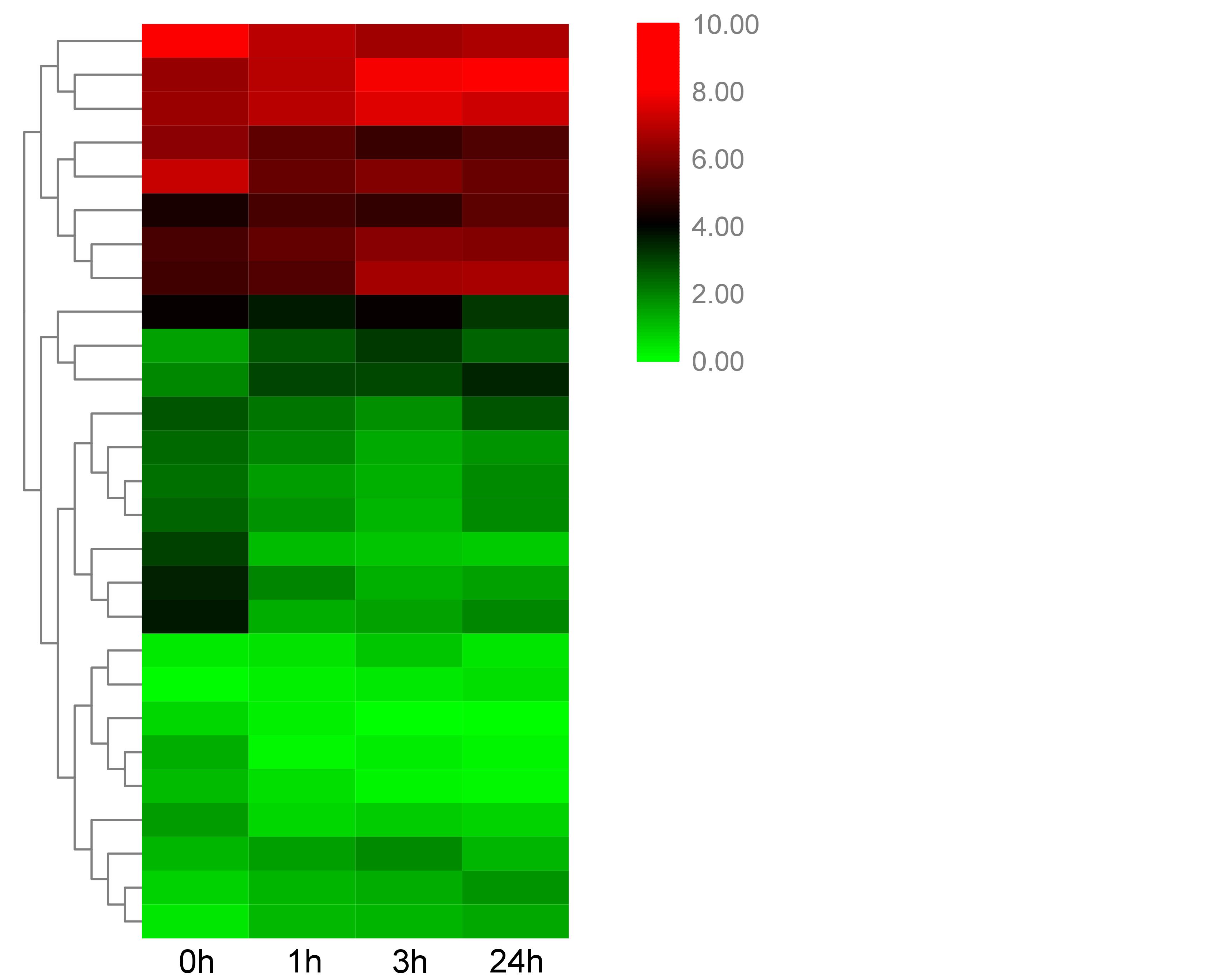

Supplement: Supplementary file 1 [file ijms-20-02771-s001.zip › 5.29 addition file/Figure S5_Heat map of DEGs enriched in Transport and catabolism.xlsx.jpg]

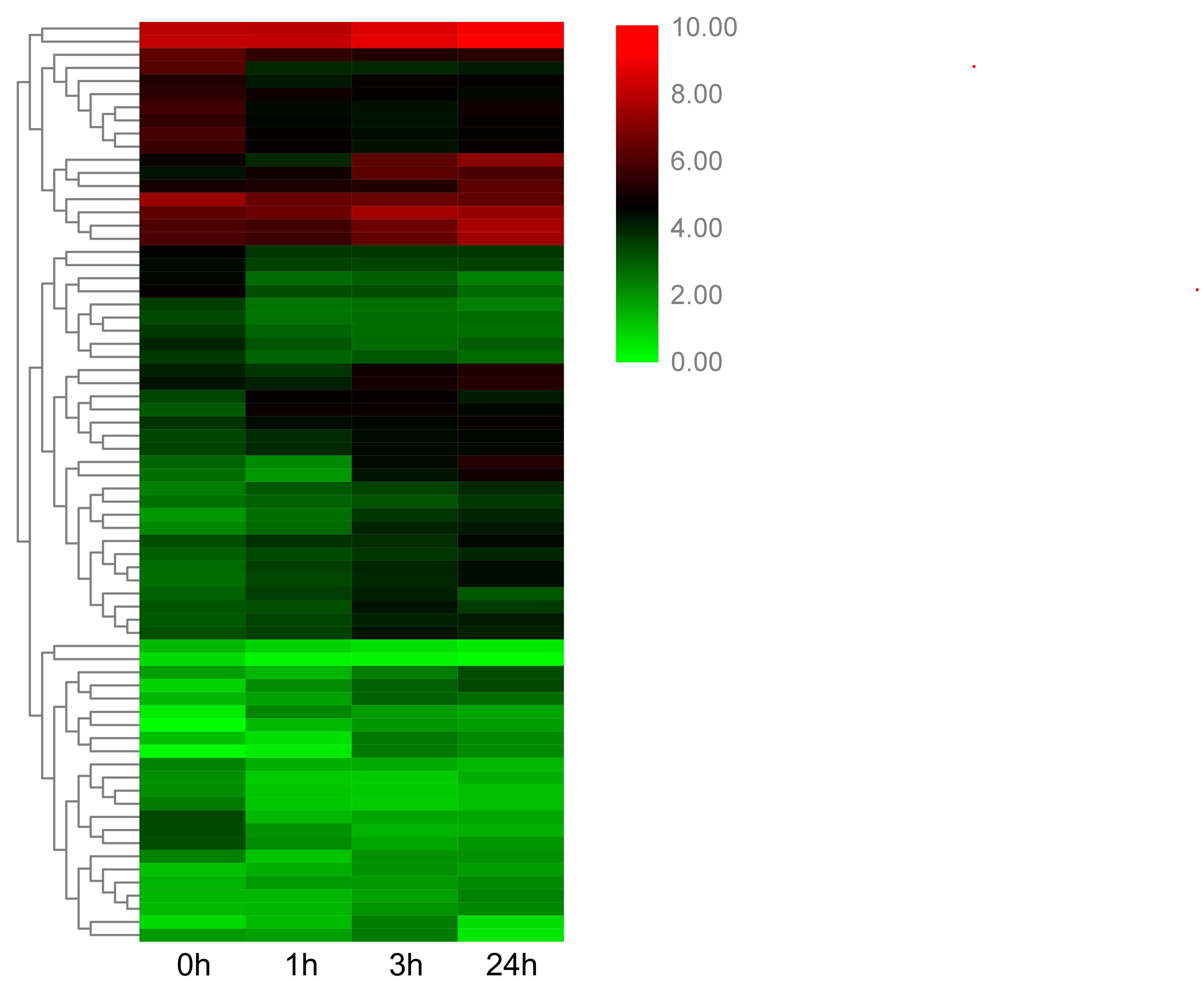

Supplement: Supplementary file 1 [file ijms-20-02771-s001.zip › 5.29 addition file/Figure S6_Hea tmap of DEGs enriched in Biosynthesis of other secondary metabolites.xlsx.jpg]

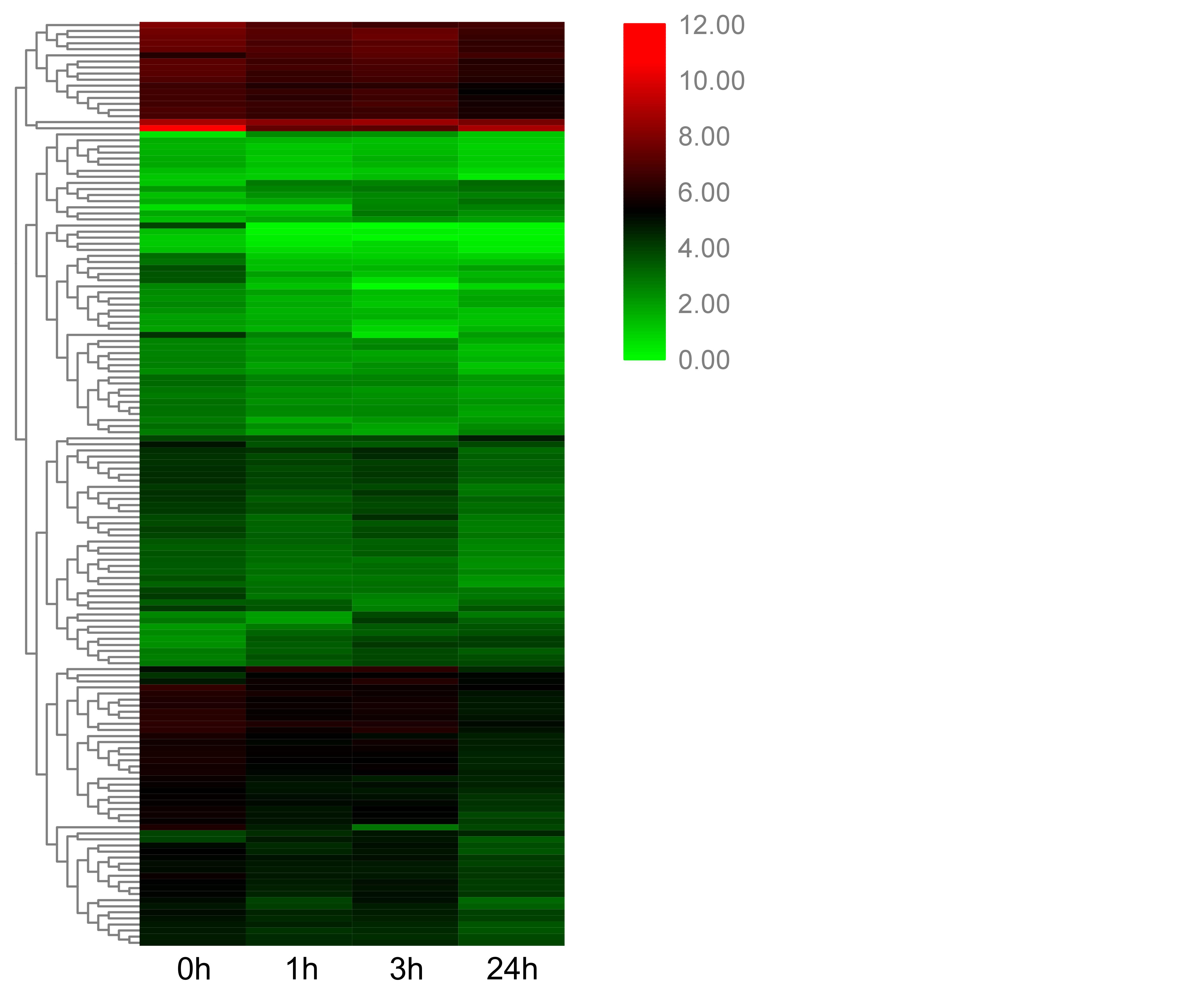

Supplement: Supplementary file 1 [file ijms-20-02771-s001.zip › 5.29 addition file/Figure S7_Heat map of DEGs enriched in Carbohydrate metabolism.xlsx.jpg]

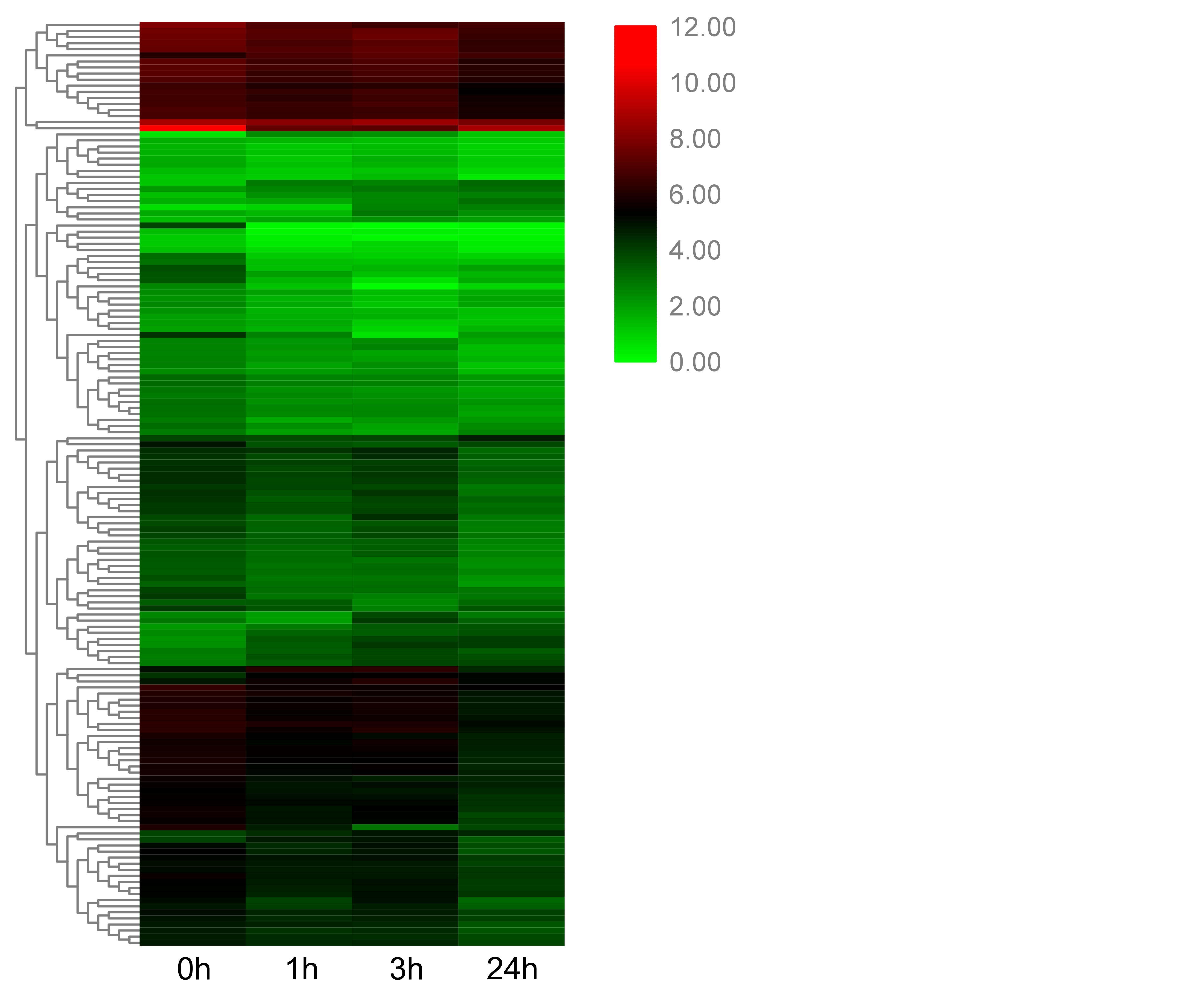

Supplement: Supplementary file 1 [file ijms-20-02771-s001.zip › 5.29 addition file/Figure S8_Heat map of DEGs enriched in genetic information processing.xlsx.jpg]

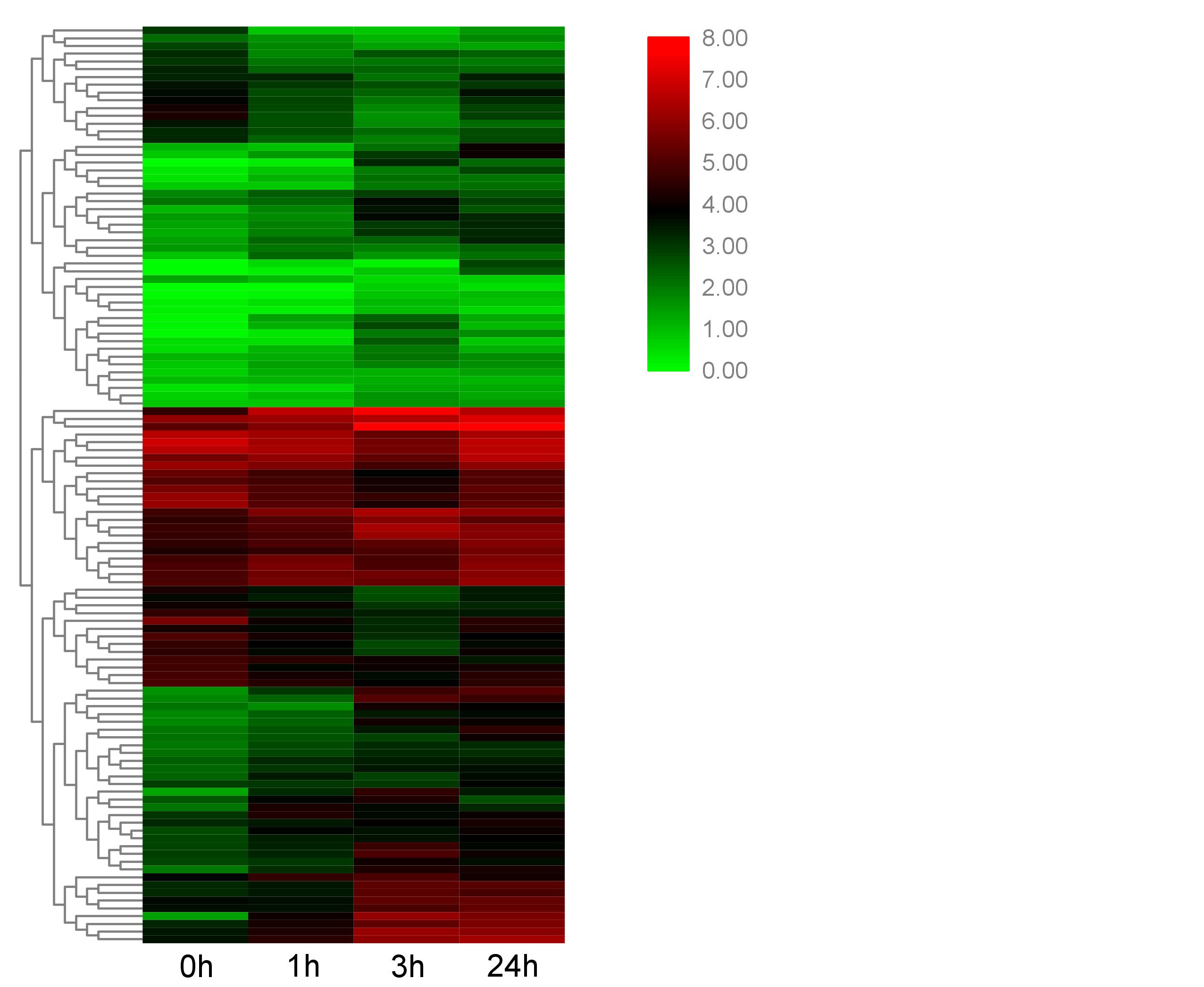

Supplement: Supplementary file 1 [file ijms-20-02771-s001.zip › 5.29 addition file/Figure S9_Heat map of DEGs enriched in Signal transduction.xlsx.jpg]
